# Transient Occlusion of Bilateral Internal Iliac Arteries Facilitates Bloodless Operative Field in Subcapsular Prostatectomy

**DOI:** 10.1155/2012/812615

**Published:** 2012-02-16

**Authors:** Takumi Takeuchi, Masayoshi Zaitsu, Koji Mikami, Shunsuke Yui, Yuta Takeshima, Naohiko Okamoto, Sadao Imao

**Affiliations:** Department of Urology, Kanto Rosai Hospital, 1-1 Kizukisumiyoshi-cho, Nakahara-ku, Kawasaki 211-8510, Japan

## Abstract

Transurethral resection of the prostate is the gold standard of surgical treatment for benign prostatic hyperplasia (BPH). Nevertheless, open subcapsular prostatectomy is still performed for large BPH. While enucleation of prostatic adenoma is being performed, unneglectable bleeding can occur and surgeons need to rush to remove adenomas, often using fingers and in a blinded fashion. The blood supply to the prostatic capsule and adenoma can be reduced to a marked extent in subcapsular prostatectomy if the bilateral internal iliac arteries are transiently occluded. Thus, a bloodless operative field is reasonably acquired during enucleation of adenoma, which would, otherwise, be a cause for concern to surgeons due to bleeding. It is not always applicable, but it could be an option if the estimated volume of BPH is more than 100 mL. In two cases, bilateral internal iliac arteries were occluded with Bulldog clamps, and then adenomas of 159 and 97 g were enucleated.

## 1. Introduction

Transurethral resection of the prostate is the gold standard of surgical treatment for benign prostatic hyperplasia (BPH). Nevertheless, open subcapsular prostatectomy is still performed for large BPH. While enucleation of prostatic adenoma is being performed, unneglectable bleeding can occur and surgeons need to rush to remove adenomas, often using fingers and in a blinded fashion. Urologic surgeons transiently occlude the renal artery when they perform partial nephrectomy in order to reduce bleeding during manipulation of the kidney; thus, it is quite logical to occlude the internal iliac artery during manipulation of the prostate. Similarly, the Pringle maneuver by clamping of the hepatic pedicle containing the hepatic artery and portal vein is employed to reduce vascular inflow during hepatic parenchymal transaction to minimize blood loss during hepatic surgery [1]. Here, we show a convenient technique to reduce bleeding and prepare a bloodless operative field by transiently occluding the bilateral internal iliac arteries during the enucleation of large prostatic adenomas.

## 2. Case Series of Subcapsular Prostatectomy

**Figure 1:**
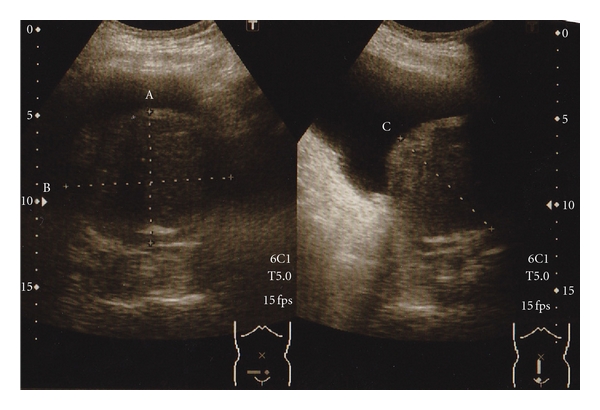
Transabdominal ultrasonography of large BPH in Case 1.

**Figure 2:**
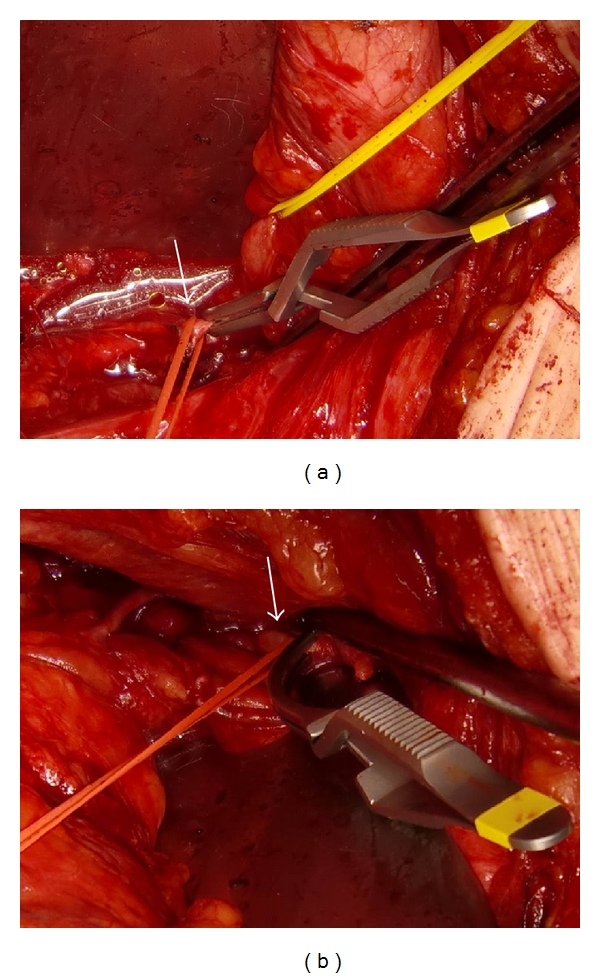
Photograph showing occlusion of bilateral internal iliac arteries by Bulldog clamps. (a) Clamping of right internal iliac artery, (b) Clamping of left internal iliac artery (white arrows).

**Figure 3:**
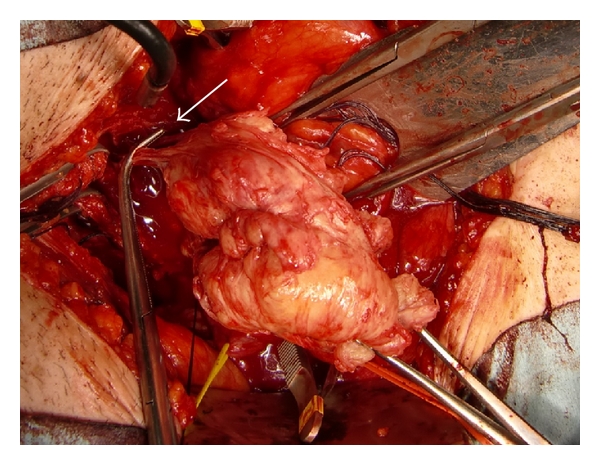
Clamping of vessels flowing into prostatic adenoma under clear vision (white arrow).

**Figure 4:**
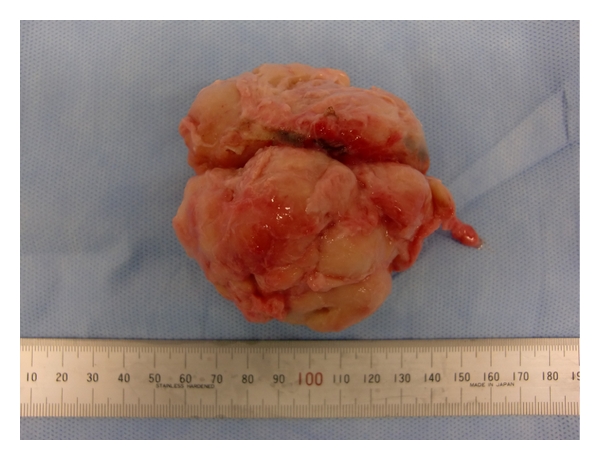
Enucleated adenoma weighing 159 g in Case 1.

In Case 1, BPH of 160 g estimated by transabdominal ultrasonography (Figure 1) was determined to be removed by open subcapsular prostatectomy. Bilateral internal iliac arteries were isolated and taped following a lower midline skin incision. Then, two lines of sutures with absorbable strings were applied to the prostatic capsule. After the bilateral internal iliac arteries were occluded with Bulldog clamps (Figure 2), the prostatic capsule was incised between the lines of sutures. Despite the quite large size of the prostatic adenoma to be enucleated, there was little bleeding until the prostatic adenoma was removed. The prostatic adenoma was enucleated and the urethra was cut with scissors, while prostatic vessels at 5 o'clock and 7 o'clock were ligated and cut under clear vision (Figure 3). Finally, a prostatic adenoma of 159 g was removed (Figure 4). The Bulldog clamps were removed before closing incision of the prostatic capsule, then hemostasis was secured. The occlusion time of internal iliac arteries was 90 minutes. The bladder neck at 5 o'clock and 7 o'clock was tied with a prostatic bed and the incised prostatic capsule was approximated with absorbable sutures. A three-way urethral catheter was placed and the balloon was inflated to 50 mL. The bladder was continuously irrigated with saline and the urethral catheter was tracted with 500 g for several hours. Blood transfusion was completely unnecessary although the exact amount of bleeding could not be evaluated, because the suction fluid was a mix of urine and blood. The postoperative course was uneventful. Pre- and postoperative hematocrit values were 40.7 and 31.9%, respectively.

In Case 2, the procedure was essentially similar, with the enucleated adenoma weighing 97 g, while the result of preoperative evaluation of the prostate volume by transabdominal ultrasonography was 121 g. The occlusion time of internal iliac arteries was 62 minutes. Blood transfusion was unnecessary, and the postoperative course was uneventful. The most important thing was that surgeons remained calm and did not need to rush to remove adenoma.

## 3. Discussion

Bilateral ligation of the internal iliac arteries has often been used in order to control massive bleeding in the field of obstetric emergency [2]. In urologic surgery, this maneuver is not clearly documented in the medical literature. Interventional embolization of bilateral internal iliac arteries is conducted for uncontrollable bladder or prostate bleeding [3]. We have controlled massive bladder bleeding in a case of severe, potentially life-threatening hemorrhagic cystitis possibly caused by an anticancer drug, melphalan, by ligation of the bilateral internal iliac arteries [4].

When performing retropubic radical prostatectomy for localized prostate cancer, urologic surgeons may think that abrupt, massive bleeding from the dorsal vein complex and neurovascular bundles aside the urethra is very rare in the antegrade approach compared to the retrograde one, probably depending on the direction of separating the prostate from surrounding tissues. It may be because the blood supply to the prostate and penis as well, which mostly originates from branches of internal iliac arteries [5], is markedly decreased in the process of separating the prostate in the antegrade approach till the dorsal vein complex and the neurovascular bundles are transected or separated from the prostate.

Similarly, the blood supply to the prostatic capsule and adenoma must be reduced to a marked extent in subcapsular prostatectomy if bilateral internal iliac arteries are transiently occluded. Thus, a bloodless operative field is reasonably acquired during enucleation of the adenoma, which, otherwise, concerns surgeons due to bleeding. In addition to surgeons' calmness, benefits of decreased bleeding are improved visualization in the operative fields, better surgical precision as a result, and fewer blood transfusions. It took a longer occlusion time in the first case as the adenoma was larger and surgeons had less experience with the new maneuver, but the occlusion time could be reduced with experience. Of course, it is unnecessary to always occlude internal iliac arteries in subcapsular prostatectomy, but it could be an option if the estimated volume of BPH is approximately more than 100 mL. Complications of uni- and bilateral internal iliac artery occlusion include buttock and thigh claudication, new-onset erectile dysfunction, colonic ischemia, spinal cord ischemia, and scrotal skin sloughing [6]. We have followed up our patients for three months, and these complications have not been observed. Transient occlusion of the bilateral internal iliac arteries may be performed safely.

The same maneuver is also applicable to difficult intrapelvic surgeries such as those of intrapelvic sarcoma and advanced bladder cancer in order to avoid massive bleeding. The first author has already experienced occluding and even ligating bilateral internal iliac arteries before the separation of target organs from surrounding tissues in such cases, resulting in acquiring relatively bloodless operative fields. In conclusion, transient occlusion of the bilateral internal iliac arteries effectively facilitates bloodless operative field when performing subcapsular prostatectomy.

## References

[1] Pringle JH (1908). V. Notes on the arrest of hepatic hemorrhage due to trauma. *Annals of Surgery*.

[2] Fernandez H, Pons JC, Chambon G, Frydman R, Papiernik E (1988). Internal iliac artery ligation in post-partum hemorrhage. *European Journal of Obstetrics & Gynecology and Reproductive Biology*.

[3] Delgal A, Cercueil JP, Koutlidis N (2010). Outcome of transcatheter arterial embolization for bladder and prostate hemorrhage. *The Journal of Urology*.

[4] Takeuchi T, Zaitsu M, Mikami K (2010). A case of severe hemorrhagic cystitis caused by melphalan with successful bladder preservation by ligation of bilateral internal iliac arteries. *Case Reports in Medicine*.

[5] Clegg EJ (1955). The arterial supply of the human prostate and seminal vesicles. *Journal of Anatomy*.

[6] Rhodes K, Didomenico P, Vatakencherry G (2011). Bilateral internal iliac artery occlusion for EVAR. *Vascular Disease Management*.

